# Effects of ZnMgO Electron Transport Layer on the Performance of InP-Based Inverted Quantum Dot Light-Emitting Diodes

**DOI:** 10.3390/nano11051246

**Published:** 2021-05-09

**Authors:** Binbin Zhang, Yu Luo, Chaohuang Mai, Lan Mu, Miaozi Li, Junjie Wang, Wei Xu, Junbiao Peng

**Affiliations:** State Key Laboratory of Luminescent Materials and Devices, Institute of Polymer Optoelectronic Materials and Devices, South China University of Technology, Guangzhou 510640, China; msbinbinzhang@foxmail.com (B.Z.); luoyulucas@163.com (Y.L.); 201710102477@mail.scut.edu.cn (C.M.); b11010904ml@163.com (L.M.); msmzli@mail.scut.edu.cn (M.L.); wangjunjie20200928@163.com (J.W.); psjbpeng@scut.edu.cn (J.P.)

**Keywords:** Mg-doped zinc oxide, InP, QLED, electron transport layer, energy band

## Abstract

An environment-friendly inverted indium phosphide red quantum dot light-emitting diode (InP QLED) was fabricated using Mg-doped zinc oxide (ZnMgO) as the electron transport layer (ETL). The effects of ZnMgO ETL on the performance of InP QLED were investigated. X-ray diffraction (XRD) analysis indicated that ZnMgO film has an amorphous structure, which is similar to zinc oxide (ZnO) film. Comparison of morphology between ZnO film and ZnMgO film demonstrated that Mg-doped ZnO film remains a high-quality surface (root mean square roughness: 0.86 nm) as smooth as ZnO film. The optical band gap and ultraviolet photoelectron spectroscopy (UPS) analysis revealed that the conduction band of ZnO shifts to a more matched position with InP quantum dot after Mg-doping, resulting in the decrease in turn-on voltage from 2.51 to 2.32 V. In addition, the ratio of irradiation recombination of QLED increases from 7% to 25% using ZnMgO ETL, which can be attributed to reduction in trap state by introducing Mg ions into ZnO lattices. As a result, ZnMgO is a promising material to enhance the performance of inverted InP QLED. This work suggests that ZnMgO has the potential to improve the performance of QLED, which consists of the ITO/ETL/InP QDs/TCTA/MoO_3_/Al, and Mg-doping strategy is an efficient route to directionally regulate ZnO conduction bands.

## 1. Introduction

In the past decade, quantum dot (QD) has been considered a promising next-generation light-emitting material due to its outstanding photo-electrical properties [1,2,3] and great solution-processing ability [4,5]. Among well-documented QD materials, cadmium (Cd)-based QDs have tremendous potential in commercial application of displays [6,7,8] because of their high external quantum efficiency (EQE) [9] and excellent luminescence performance [10]. However, with the increase in people’s awareness of environmental protection, the toxicity of Cd-based QDs has attracted more and more attention [11,12,13], which limits the application of Cd-based QDs.

Developing eco-friendly, low-toxic or non-toxic QD materials is key to solving the above problems [14]. Indium phosphide (InP)-based QDs have attracted wide attention from researchers because of their high fluorescence quantum yield, low toxicity, and environment friendliness [15,16,17], and are considered a candidate to replace Cd-based QDs [18]. Generally, a multi-layer device structure that includes an electron injection/transport layer (EIL/ETL), emitting layer (EML), hole injection/transport layer (HIL/HTL), and electrode layer is required to achieve high-performance InP-based QLED, which helps to balance the injection of carriers and reduce the barrier of interface [1,19,20,21]. Among these layers, the ETL plays an important role in electron injection efficiency, exciton dissociation, and hole blocking [22,23,24]. A well-known and widely applied ETL material is zinc oxide (ZnO), which has good stability and high electron mobility [5,24,25]. However, many challenges still exist for ZnO application in InP QLED, such as surface defects, work function mismatching, and so forth, which may cause the quenching of excitons and interface barriers. The doping strategy is an efficient route to optimizing the performance of ZnO ETL. Sun et al. achieved a 36% improvement in external quantum efficiency (EQE) in comparison with conventional ZnO/QD devices using MgZnO-modified ETL [26]. Their device’s current efficiency was enhanced by inserting MgZnO between ZnO and quantum dots, but the device’s turn-on voltage increased after introducing MgZnO. Moon et al. developed a tailored ZnMgO layer that was applied in green InP QLED, and a maximum luminance of 13,900 cd/m^2^ and an external quantum efficiency (EQE) of 13.6% were realized for the first time [27]. However, their studies focused on the conventional InP QLEDs with a structure ITO/PEDOT: PSS/poly-TPD: PVK/InP/ZnMgO/Al. Li et al. found that an increasing Mg-doping level can broaden the ZnO band gap, shift its energy levels, and, most importantly, increase its resistivity. They achieved much more balanced charge injection and realized high efficiency (11.6 cd/A) in red conventional InP QLED devices [28]. In general, an inverted device structure of QLED is preferable for QLED in the application field of active matrix QLED display because the indium tin oxide (ITO) electrode of the active matrix panel can directly act as the cathode of QLED, helping to reduce the driving voltage and enhance the stability of QLED devices [29,30]. Based on the reported studies, we found that some questions remain to be answered: (1) Are the advantages of ZnMgO ETL general for QLED with various material systems? (2) For an inverted QLED structure, what are the effects of ZnMgO ETL on device performance?

In this work, we introduced the ZnMgO nanoparticles as the ETL in InP-based red QLED with an inverted device structure. The effects of Mg-doping on the component, phase, morphology, and energy level structures of ZnMgO film were investigated. The performance of ZnMgO-based InP red QLED was analyzed. We verified that introduction of ZnMgO ETL can still improve the performance of InP QLED devices with an inverted structure. In addition, we found that Mg-doping can broaden the band gap of ZnO, which is mainly attributed to the conduction band minimum (CBM) shift upward. The optimizing of band structure and reducing of traps and defects result in improvements in turn-on voltage and luminance properties.

## 2. Experimental Method

### 2.1. Materials

The ITO glass substrates were purchased from China Southern Glass Holding Corp. (Shenzhen, China) with sheet resistance around 17 Ω/sq. ZnO and ZnMgO nanoparticle solution was purchased from Guangdong Poly OptoElectronics Corp. (Jiangmen, China), with a concentration of 20 mg/mL in ethanol. The indium phosphide quantum dots (InP QDs) were purchased from Suzhou Xingshuo Nanotech Corp. (Suzhou, China), with the concentration of 25 mg/mL in normal octane. The 4,4′,4′′-tris(carbazol-9-yl) triphenylamine (TCTA) was purchased from Jilin OLED Material Tech Corp. (Changchun, China). The molybdenum oxide (MoO_3_) was purchased from Sigma Aldrich (St. Louis, MO, USA).

### 2.2. Device Fabrication

Clean indium tin oxide (ITO) substrate was irradiated by UV light for 10 min to improve surface wettability. The InP QLED was fabricated with an inverted structure, consisting of the ITO/ZnO or ZnMgO/InP QDs/TCTA/MoO_3_/Al. First, the ZnO or ZnMgO ETL was spin-coated at 3000 rpm for 30 s (thickness: ~40 nm). The as-deposited ETL was annealed at 120 °C for 12 min. Then the QDs layer was deposited on the ETL spin-coating at 3000 rpm for 30 s and annealed at 120 °C for 10 min (thickness: ~34 nm). The TCTA, MoO_3_ and Al electrodes were prepared by thermal evaporation process in turn, whose thickness was 60 nm, 7 nm and 110 nm, respectively. All processes were conducted in the glove box for isolating water and oxygen.

### 2.3. Characterization

The thickness of ETL and QDs was measured by a step profiler (Bruker Dektak XT, Karlsruhe, Germany). The photoluminescence (PL) spectra were determined using a QE Pro spectrometer (Biaoqi Optoelectronics, Guangzhou, China). The phase of ZnO or ZnMgO ETL was analyzed by X-ray diffraction (XRD, Panalytical, Malvern, UK). The ratio and chemical state of elements were determined by X-ray photoelectron spectroscopy (XPS, Thermo Fisher Scientific, Waltham, MA USA, K-ALPHA+, Source: Al Kα). The current density (J), voltage (V), and luminance (L) characteristics were measured using a Keithley (Cleveland, OH, USA) 2400 source meter and a Konica Minolta (Tokyo, Japan) Chroma Meter CS-200. Film morphology was acquired by atomic force microscope (AFM, Bruker Multimode 8, Karlsruhe, Germany) and a scanning electron microscope (SEM, Zeiss Merlin, Oberkochen, Germany). Electroluminescence spectra were recorded using a photometer (PhotoResearch PR-705, Los Angeles, CA, USA). Time-resolved photoluminescence (TRPL) spectra were recorded using C11367-11 (Hamamatsu Photonics, Iwata, Japan). Absorption spectra were obtained by UV-2600 (SHIMADZU, Kyoto, Japan), and the optical band gap was further calculated using the absorption spectra. Valence band maximum was analyzed by ultraviolet photoelectron spectroscopy (UPS, Thermo Fisher Scientific, Waltham, MA, USA, K-ALPHA+, Source: He I).

## 3. Results and Discussion

**Figure 1:**
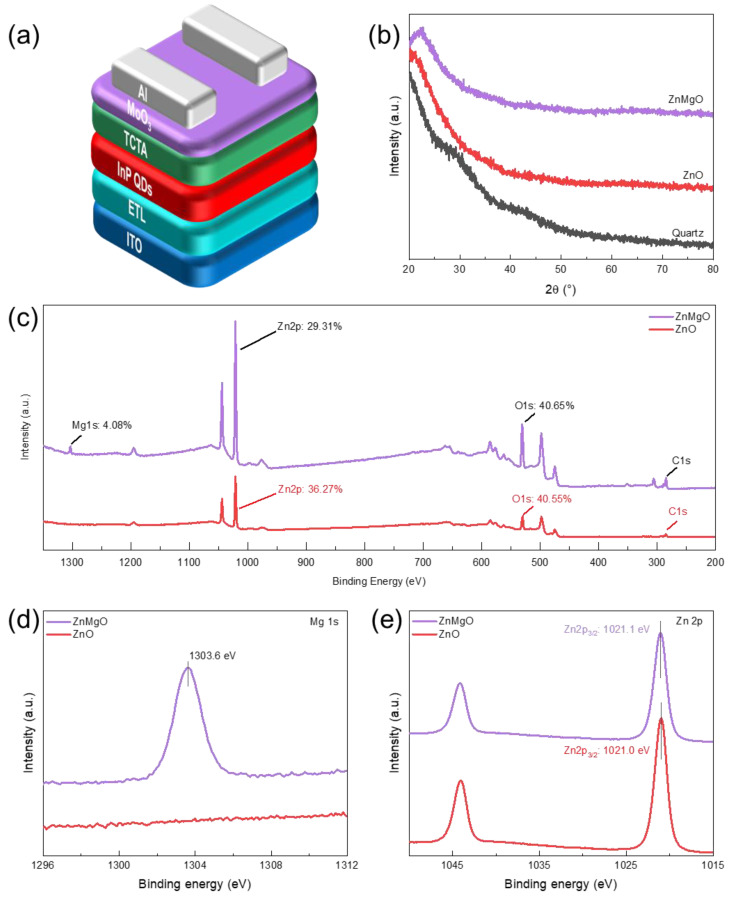
(**a**) Schematic diagram showing the structure of the device; (**b**) XRD patterns of the ZnO and ZnMgO films; (**c**) full spectra of XPS, and the ratios of Mg, Zn, and O elements were calculated using the peak areas; (**d**) fine XPS spectra of Mg element; and (**e**) fine XPS spectra of Zn.

Figure 1a shows the basic device structure of the inverted InP QLED. Figure 1b shows that there is no identifiable diffraction peak of ZnO and ZnMgO film in a wide 2θ range, indicating that both the ZnO and ZnMgO film are amorphous. Next, the elements of ZnO and ZnMgO film were determined by XPS (Figure 1c). The ratios of main elements Zn, Mg, and O are represented using the area ratio of their respective peak areas to the total area in the full XPS spectrum. In the ZnMgO film, the ratio of Mg and Zn is 4.08% and 29.31%, respectively, meaning that the molar doping ratio of Mg is around 12.2% (Mg/(Mg + Zn) × 100%), which is basically consistent with the reported optimal molar doping ratio (12.5%) of ZnMgO [27]. Figure 1d shows the fine XPS spectra of Mg. The peak of Mg 1s located at 1303.6 eV can be observed in the spectrum of the ZnMgO film, and deviates from the peak of Mg (1303.3 eV) [31], which indicates that Mg loses the outer electrons. Further, in Figure 1e, the binding energy of Zn 2p_3/2_ in ZnMgO (1021.1 eV) is higher than that of Zn 2p_3/2_ in ZnO (1021.0 eV), revealing that the Zn-O-Mg bonds are formed in ZnMgO film [27]. The successful alloying of Mg and ZnO provides a physical basis for regulating the band structure of ZnMgO because the Mg ions have fewer electrons and simpler energy level structure than Zn ions.

**Figure 2:**
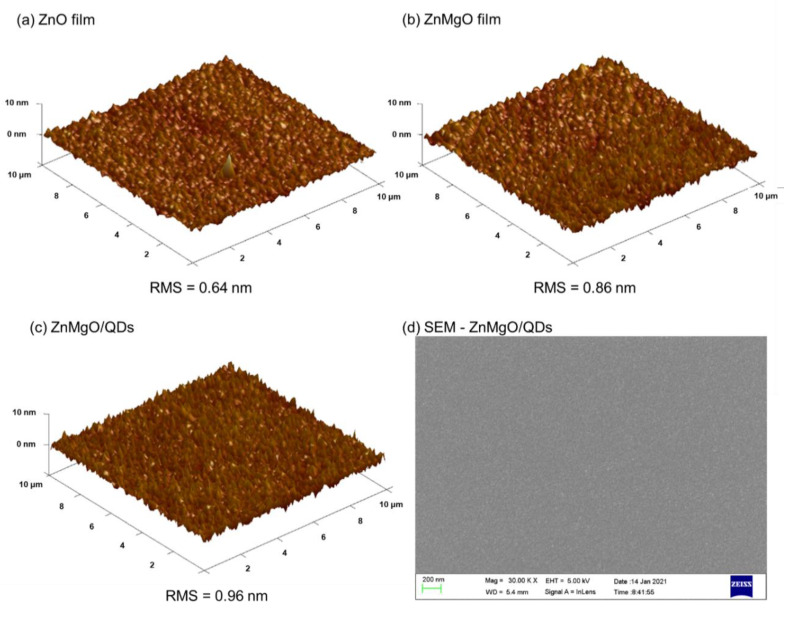
The 3D AFM images of (**a**) ZnO film, (**b**) ZnMgO film, and (**c**) ZnMgO/QDs multilayer surface; (**d**) SEM image of ZnMgO/QDs multilayer surface.

To investigate the effects of Mg-doping on the film surface, an AFM measurement was carried out for the ZnO and ZnMgO films (Figure 2). Figure 2a,b shows that there are few pores in the ZnO and ZnMgO film, helping to reduce the defects that influence carrier transfer. The root mean square roughness (RMS) of ZnMgO film (0.86 nm) is slightly higher than that of ZnO film (0.64 nm), which may be attributed to the lattice distortion caused by the various sizes of Mg and Zn ions. In addition, both the ZnO and ZnMgO films show few pores and particles on the surface, indicating that they are amorphous (Figure 1a). The InP QDs layer was spin-coated on the top of ZnMgO film, whose AFM image (Figure 2c) also demonstrates that the surface of ZnMgO/QDs multilayer structure remains poreless, particle-less, and smooth (0.96 nm). The SEM image (Figure 2d) further indicates that InP QDs are distributed uniformly, and the surface properties (smooth, particle-less, poreless, etc.) are similar to the results of AFM. The smooth surface plays a vital role in reducing defects between layers, which helps to transport carriers and to reduce the nonradiative recombination at the interface. The result of the morphology investigation illustrated that the Mg-doping does not cause interface deterioration.

**Figure 3:**
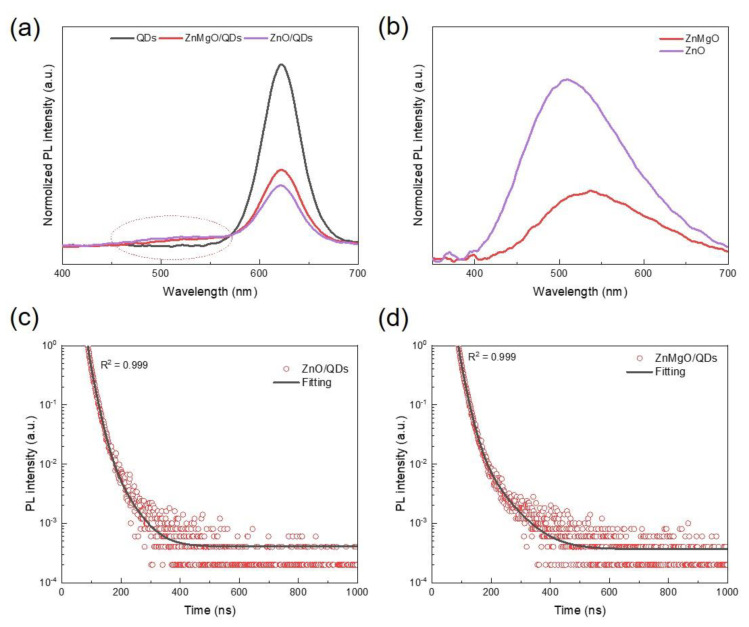
(**a**) PL intensity of QDs, ZnMgO/QDs, and ZnO/QDs films; (**b**) PL intensity of ZnMgO and ZnO films; (**c**) TRPL spectra of the QDs using ZnO ETL; and (**d**) TRPL spectra of the QDs using ZnMgO ETL. The solid lines are the fitting curves through a three-exponential decay model.

The effects of ZnMgO ETL on the PL property of InP QDs were studied (Figure 3a). As can be seen in Figure 3a, the PL intensity of ZnMgO/QDs is higher than that of ZnO/QDs. A PL peak at around 530 nm is observed in the PL spectra of ZnMgO/QDs and ZnO QDs, implying that both ZnMgO and ZnO have defective luminescence. To compare the intensity of defective luminescence, another PL spectra measurement for ZnO layer and ZnMgO layer was conducted (Figure 3b). It was demonstrated that the ZnMgO layer has less defective luminescence, which explains why the PL intensity of ZnMgO/QDs is higher than that of ZnO/QDs.

In addition, a measurement of TRPL spectra for ZnMgO/QDs and ZnO/QDs was conducted to quantitatively evaluate the effect of defects on luminescence. Figure 3c,d shows the TRPL results and their fitting curves through a three-exponential decay model as follows [30]:

(1) $$A\left(t\right)=A_{1}\exp\left(-\frac{t}{\tau_{1}}\right)+A_{2}\exp\left(-\frac{t}{\tau_{2}}\right)+A_{3}\exp\left(-\frac{t}{\tau_{3}}\right)$$

(2) $$\tau_{avg}=\frac{A_{1}\tau_{1}^{2}+A_{2}\tau_{2}^{2}+A_{3}\tau_{3}^{2}}{A_{1}\tau_{1}+A_{2}\tau_{2}+A_{3}\tau_{3}}$$

(3) $$P_{i}=\frac{A_{i}\tau_{i}}{A_{1}\tau_{1}+A_{2}\tau_{2}+A_{3}\tau_{3}}\times 100\%$$

In Equation (1), the exponential items of $A_{1}$, $A_{2}$, and $A_{3}$ represent the three kinds of exciton recombination process, and their respective decay lifetimes are $\tau_{1}$, $\tau_{2}$, and $\tau_{3}$. The average decay lifetime is defined by Equation (2). Then, the ratio of each exciton recombination process was calculated using Equation (3). Based on the results of three-exponential decay fitting, the fitting parameters are listed in Table 1.

Among the three decay lifetimes of exciton recombination process, the long lifetime ($\tau_{1}$) and the middle lifetime ($\tau_{2}$) are from the two kinds of trap-assisted recombination [19], and the short lifetime ($\tau_{3}$) is attributed to the recombination process of electron and hole that happens in lattice, which represents the decay lifetime of radiative recombination [19,30]. As shown in Table 1, the ratio of radiative recombination increases from 7% to 25% when the ZnMgO acts as the ETL.

**Figure 4:**
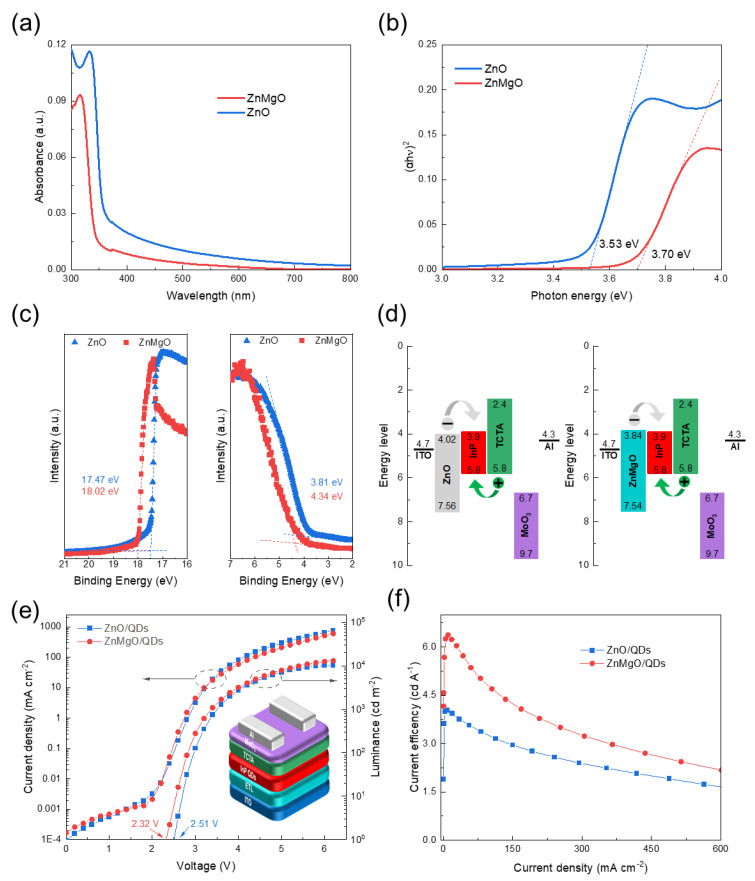
(**a**) Absorption of ZnO and ZnMgO films in a UV-visible range; (**b**) optical band gaps of ZnO and ZnMgO films; (**c**) UPS spectra of ZnO film and ZnMgO film; (**d**) band diagrams of the QLED with ZnO ETL and ZnMgO ETL; (**e**) J-V-L curves of inverted InP QLED with different ETL. The inset films showing the structure of device. (**f**) The dependence of current efficiency on current density.

The band structure of ZnMgO and ZnO was investigated to understand the effects of Mg-doping on the position of the valence band and conduction band. Figure 4a shows the absorption spectra of ZnMgO and ZnO in a range of 300–800 nm. The absorption edge of ZnMgO is located at a shorter wavelength than that of ZnO. In addition, the optical band gap (*E_g_*) of ZnMgO and ZnO was determined using the absorption spectra [32,33]. The Tauc plot can be drawn by following the Equation (4) [28,32,33,34]:

*αhν = A(hν-E_g_)^n^* (4)

where α represents the absorption coefficient, *hν* is the photon energy that is calculated by the wavelength, *A* is a constant, and the value of *n* is 1/2 for the direct bandgap semiconductor of ZnMgO. The horizontal intercept of *(αhν)^2^* versus photon energy curves represents the *E_g_*. Figure 4b indicates that the *E_g_* of ZnMgO (3.7 eV) is higher than that of ZnO (3.53 eV).

UPS measurement was used to determine the valence band of ZnO and ZnMgO, and the result is depicted in Figure 4c. In Figure 4c, the high binding energy cutoff edges of ZnO and ZnMgO are 17.47 and 18.02 eV, respectively; and the low binding energy cut-off edges are 3.81 and 4.34 eV, respectively, which establish the valence band maximum values of ZnO and ZnMgO at 7.56 and 7.54 eV, respectively [35]. In addition, the conduction band minimum values of ZnO and ZnMgO can be calculated using their valence band maximum value and *E_g_* value, which are 4.02 eV and 3.84 eV, respectively. The HOMO and LUMO of InP QDs were also measured (Supplementary Materials, Figure S1). Figure S1a shows the UV-visible spectra of InP QDs, revealing its *E_g_* is 1.9 eV. Figure S1b indicates the HOMO value is 5.8 eV, and the LUMO is 3.9 eV. Then, the band structures of ZnO/InP-based and ZnMgO/InP-based QLED are compared in Figure 4d. Figure 4d shows the band diagrams of QLEDs, illustrating that the ZnMgO provides a more matched band structure for electron injected from ETL to QDs. Theoretically, the barrier between ETL and QDs decreases from 0.12 to −0.06 eV when ZnMgO replaces the ZnO as an ETL, which helps to reduce the turn-on voltage of QLED.

To verify this, the J-V-L curves of InP QLEDs with different ETL were recorded (Figure 4e). The inverted structure of the device is shown in the inset diagram in Figure 4e. The J-V-L results indicate that the turn-on voltage of ZnMgO/InP-based QLED is 2.32 V, and is 0.19 V less than that of ZnO/InP-based QLED, which is consistent with the theoretical analysis. Additionally, Figure 4f shows that the maximum current efficiency of ZnMgO/InP-based QLED (6.38 cd A^−1^) is higher than that of ZnO/InP-based QLED (4.03 cd A^−1^). The ZnMgO/InP-based QLED also realizes more than 1.3 × 10^4^ cd m^−2^ maximum brightness, whereas the maximum brightness of ZnO/InP-based QLED is only 1.0 × 10^4^ cd m^−2^.

## 4. Conclusions

An environment-friendly inverted InP QLED was fabricated using ZnMgO as electron transport layer. Element analysis demonstrated that Mg was alloyed into the ZnO lattice. The amorphous Mg-doping ZnO film retains a high-quality surface (RMS: 0.86 nm) as smooth as that of ZnO film, resulting in fewer traps or defects in InP QLED, which helps to enhance the irradiation recombination of QLED. In addition, we revealed that Mg-doping can broaden the band gap of ZnO, which is mainly attributed to the conduction band minimum (CBM) shift upward through band structure analysis. The enhancement in CBM level can reduce the barrier between ETL and QDs, resulting in the decrease in turn-on voltage from 2.51 to 2.32 V (reducing 0.19 V), which is basically consistent with the offset (0.18 V) of CBM after Mg-doping. The more matched energy band and fewer defects enhance the luminance property and current efficiency using ZnMgO ETL. This work verifies that the introduction of ZnMgO ETL can improve the performance of InP QLED devices with an inverted structure and suggests that the Mg-doping strategy is an efficient route to directionally regulate ZnO conduction bands. It provides a theoretical understanding for the optimization mechanism of inverted QLED’s performance using ZnMgO as ETL.

## Figures and Tables

**Table 1:** Fitting parameters for various PL decays of ZnO/QDs and ZnMgO/QDs.

	*P_1_*	*P_2_*	*P_3_*	*τ_1_* (ns)	*τ_2_* (ns)	*τ_3_* (ns)	*τ_avg_* (ns)
ZnO/QDs	18%	75%	7%	48.48	16.56	4.18	21.62
ZnMgO/QDs	11%	64%	25%	60.02	19.25	7.86	21.77

## Data Availability

Not applicable.

## Supplementary Materials

The following are available online at https://www.mdpi.com/article/10.3390/nano11051246/s1, Figure S1: InP QDs band gap and valence band maximum spectrum.
