# Supplementary material for: Effects of ZnMgO Electron Transport Layer on the Performance of InP-Based Inverted Quantum Dot Light-Emitting Diodes
Source: Nanomaterials (Basel). 2021 May 9;11(5):1246. doi: 10.3390/nano11051246 (PMC8151885; doi:10.3390/nano11051246)
Supplement: Supplementary file 1 [file nanomaterials-11-01246-s001.zip › nanomaterials-1183610-supplementary.pdf]

Supplementary Materials

Effects of ZnMgO Electron Transport Layer on the Performance of InP-Based Inverted Quantum Dot Light-Emitting Diodes

Binbin Zhang, Yu Luo, Chaohuang Mai, Lan Mu, Miaozi Li, Junjie Wang, Wei Xu * and Junbiao Peng

Institute of Polymer Optoelectronic Materials and Devices, State Key Laboratory of Luminescent Materials and Devices, South China University of Technology, Guangzhou 510640, China; msbinbinzhang@foxmail.com (B.Z.); luoyulucas@163.com (Y.L.); 201710102477@mail.scut.edu.cn (C.M.); b11010904ml@163.com (L.M.); msmzli@mail.scut.edu.cn (M.L.); wangjunjie20200928@163.com (J.W.); psjbpeng@scut.edu.cn (J.P.)

* Correspondence: xuwei@scut.edu.cn

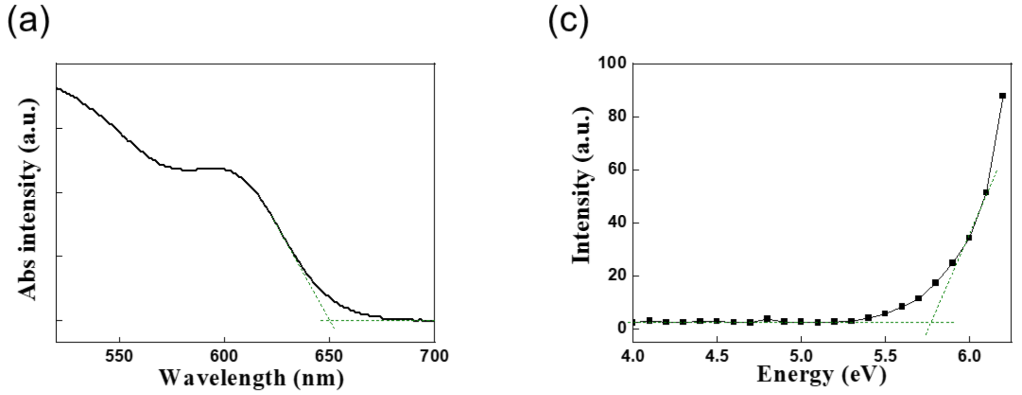

Figure S1. (a) UV-visible spectra of InP QDs, and the calculated E_g value is 1.9 eV; (b) UPS spectra of InP QDs, and the calculated HOMO and LUMO value is 5.8 eV and 3.9 eV, respectively.
